# Altruism, Scepticism, and collective decision-making in foreign-born U.S. residents in a tuberculosis vaccine trial

**DOI:** 10.1186/s12889-018-5460-3

**Published:** 2018-04-23

**Authors:** Sienna R. Craig, Timothy Lahey, Apoorva Dixit, C. Fordham von Reyn

**Affiliations:** 1 Department of Anthropology, Dartmouth College, 6047 Silsby Hall, Hanover, NH 03755 USA; 2 Geisel School of Medicine, Dartmouth College, Hanover, USA; 3 Dartmouth College, Hanover, USA

**Keywords:** Volunteerism, Clinical trials, Tuberculosis, Vaccine, Collective decision-making

## Abstract

**Background:**

The current vaccine against tuberculosis, BCG, is effective when given in most TB-endemic countries at birth but has diminished efficacy against pulmonary TB after 15–20 years. As a result, new booster vaccines for adolescents and adults are being developed to realize the World Health Organization target of global elimination of TB by 2035. Multiple TB candidates thus are in active clinical development.

**Methods:**

One of these, DAR-901, is advancing in human clinical trials. These clinical trials are conducted in BCG immunized adults with and without HIV infection in order to assess safety and efficacy among the people most in need of a new vaccine. A Phase I dose escalation trial of DAR-901 in BCG-immunized adults with or without HIV infection was conducted between 2014 and 2016. This offered an unusual opportunity to qualitatively examine why foreign-born adults living in the United States – a poorly studied population – decide to participate, or not, in clinical trials.

**Results:**

We conducted a qualitative study of individuals who were recruited to participate in this Phase I vaccine trial, interviewing those who agreed and declined to participate. We found diverse motivations for participation or refusal; varied understandings of tuberculosis and vaccines; and complex views about how ‘informed consent’ can be at odds with cultural understandings of power, authority, and medical decision-making. These dynamics included: knowledge (direct or indirect) of tuberculosis, a desire to be altruistic and simultaneous hopes for personal gain as well as concerns over what remuneration for participation could mean, the importance of personal relationships with care providers in shaping volunteerism, concerns over privacy, and evidence of how culture and history shape medical decision-making.

**Conclusions:**

This US-based trial, aimed at addressing a crucible global health issue, raises productive questions about the interface between altruism and scepticism regarding clinical research participation.

**Trial registration:**

NCT02063555.

## Background

The World Health Organization has targeted elimination of tuberculosis (TB) in the world by 2035 [1], a goal felt unreachable without the introduction of a new and more effective TB vaccine [2]. The current global vaccine against TB, known as BCG, is given at birth in most TB-endemic countries. BCG lowers the incidence of disseminated TB in children, and likely provides limited protection from TB later in life, but does not provide sufficient lifelong protection from TB [3]. Modelling studies indicate that global TB elimination by 2035 can only be achieved with the development of a new booster vaccine [4].

More than a dozen TB vaccine candidates are now in active clinical development [5, 6].^1^ DAR-901 is the leading TB vaccine candidate aimed at boosting the protective efficacy of BCG [7]. A Phase I dose escalation trial recently found that DAR-901 was safe, tolerable and immunogenic [8]. This study was conducted among HIV-negative and HIV-infected adults in the United States who received BCG at birth in countries where TB is endemic.

A Phase 1 vaccine study among foreign-born adults in the United States presents an unusual research opportunity. While the motivations for American citizens to participate in US-based clinical trials are well researched [9–11], as are the motivations for local populations to participate in global clinical trials [12–16], there is a relative dearth of literature that addresses study populations with unique cross-cultural understanding of both their home country and the US, as sites of clinical research and medical intervention. Research trials structured around the recruitment of foreign-born subjects are uncommon in the United States [17–19]. As a result, the motivations for foreign-born individuals to participate in clinical trials – or their decisions *not* to participate in such research – are poorly understood. The US-based DAR-901 trial presented an opportunity to learn more about volunteerism in a foreign-born population.

Instead of testing a drug or intervention on a ‘treatment naïve’ population which might not expect benefit from research outcomes [20], the DAR 901-trial was conducted in the US on individuals who reflected a global ‘target population’ of new TB vaccine development: those at risk of TB mortality and morbidity, including people living with HIV. For the HIV-infected subset of potential subjects, the trial was also conducted in a context of significant pre-existing trust between them and physician-scientists.^2^ And yet, early trial recruitment efforts remained difficult, with the rationales for eligible participants’ agreement or refusal to participate initially opaque to study staff.

To assess foreign-born individuals’ motivations to participate in a clinical trial, or not, we conducted a qualitative study of those who were both enrolled in and approached for recruitment into in the Phase I DAR-901 trial. In interviews with 18 enrolled and declined individuals, we found diverse motivations for participation or refusal; varied understandings of tuberculosis and vaccines; and complex views about how informed consent can be at odds with cultural understandings of power, authority, and medical decision-making. This US-based trial provided an unusual opportunity to address an issue that is crucial to the success of global health interventions: how altruism and scepticism influence medical decision-making regarding clinical research participation.

Motivations for participation in medical trials include altruism, the desire to contribute to science, and expectation of personal benefit [12, 20–24]. The predominant motivator can change from early to late stage trials; altruism can be supplanted in later-phase trials by subject expectation of benefit ([25, 26]: p. 112–128). Subjects may see study participation as solidifying the therapeutic bond between them and a clinician [27–29]. In US trials, the patient-physician relationship is a commonly cited motivating factor for participation, but is rarely acknowledged in the context of global trials [12, 30]. Ironically, volunteerism can compete with financial incentives as motivations to participate in clinical trials [31, 32]. This presents an ethically fraught dynamic, since financial incentives can become coercive [33–35]. The possibility that subjects can be coerced by financial incentives for study participation varies by age. Vere ([36]: p. 142) argues that risk acceptance among young subjects (i.e. college students) is high, and they can ‘deny that payment is for risk [and] assert that it is for inconvenience’ to justify participating [37, 38]. This was relevant to our study in that it was being conducted in a college town, where many potentially eligible foreign-born, BCG-vaccinated individuals would be undergraduate or graduate students.

Motivations not to participate in clinical trials are just as complicated [39]. Subject willingness to participate can be dampened by the legacy of unethical clinical research, from the Tuskegee syphilis experiments to more-recent controversies like the Development of AntiRetroviral Therapy (DART) trials in several African countries [40, 41]. Mistrust of clinical research too can be amplified by legacies of colonial medicine ([42]: p. 146–174). As research is conducted increasingly by private, for-profit and even corporate entities [20], subjects may feel potential community benefit from study participation has shifted to corporate benefit which is less motivating [43].

Commonly cited reasons for *not* volunteering for clinical studies include ‘lifestyle’ conflicts, which may include cultural or religious sensibilities that advise against subjecting the body to experimentation or invasive procedures as well as logistical conflicts with school or work schedules, etc. [21]. Family members, too, may express concern about their loved ones being ‘experimented on’ or similar perceptions of possible harm that outweigh the potential benefits [31, 44].

The complex mixture of motivations to participate, or not, in clinical research comes to a point in the informed consent process [9, 45–47]. Even the simple-seeming decision to give informed consent to participate in a clinical trial assumes a circumscribed or individualistic understanding of informed consent. This may be complicated by cultural understandings of power, authority, and decision-making as well as the scientific vocabularies, standards, and cultural assumptions behind clinical studies [48–51]. Nurses can play critical roles in recruitment [27, 52]. For instance, subjects are more likely to understand the premise, process, risks, and benefits of a clinical trial and to ask questions (and therefore exercise more truly ‘informed’ consent) if a nurse, either with a physician or instead of a physician, works with the research team to explain this concept [17, 25]. This insight is relevant to the DAR-901 trial. As we describe below, study staff played a crucial role in developing and maintaining rapport and trust – both with those who chose to enrol in the DAR-901 trial and this qualitative study and those who declined enrolment in either or both but who might also still receive their routine clinical care at this medical institution.

The present study examined which of these complex motivations predominated among foreign-born HIV-negative and HIV-infected adults asked to participate in a Phase 1 vaccine clinical trial. Speaking with such individuals is not equivalent to speaking with potential subjects in countries where TB is endemic and a new vaccine is most likely to be deployed, but our findings do point to social dynamics that may occur in such contexts. Finally, we hope this work informs the relatively under-studied arena of the lived experiences of foreign-born individuals who choose to participate in medical research in the US.

## Methods

### Phase I TB vaccine study subject recruitment and enrolment

Information about the clinical trial and participant recruitment was publicised through a variety of community events, social networks, and physician-scientist outreach to eligible patients, particularly in the case of the HIV-infected cohort. This generated an initial group of individuals who approached the study coordinator with the intent to possibly participate. The study coordinator would then meet individually for an hour with prospective participants to explain the trial and review informed consent materials that described risks and benefits of the study. This also included a detailed discussion about what participation in the trial would involve: namely, ten clinical visits over ten months and six blood draws per patient, with those who enrolled in the study offered $600 as remuneration for time and travel. After an initial screening process to determine eligibility and the in-depth consent process, 78 subjects agreed to be screened for the trial and 59 individuals were found to be eligible and were enrolled in the trial.

### Qualitative study of potential subject motivations for participation

To explore potential research subject motivations to volunteer – or not – in a Phase I TB vaccine trial, we conducted 18 in-depth, semi-structured ethnographic interviews with enrolled and declined study subjects among three foreign-born cohorts participating in the DAR-901 study.^3^ We categorized participants into three groups:

1. declined, HIV-negative
2. enrolled, HIV-negative
3. enrolled, HIV-infected

By ‘declined,’ we mean subjects who qualified to participate in the DAR-901 clinical trial and were approached by study staff with an intention to recruit, but who declined to participate. We also attempted to interview HIV-infected subjects who declined to participate in the trial (a logical fourth cohort), but of the 6 such individuals we contacted, none agreed to participate in the qualitative study either. This is both a limitation of the research and a notable result in itself.^4^

To protect confidentiality and facilitate informed decision-making, we were not put in contact with potential study participants unless they expressed an affirmative interest in the qualitative study after being appraised of its nature and goals by the DAR-901 study coordinator, with whom they were in contact throughout the DAR-901 clinical trial process. Interviews with enrolled individuals were scheduled concurrently with study visits in the DAR-901 trial, to minimize the impact of the qualitative research on participants’ time. Interviews took place in private locations without the presence of DAR-901 study staff. Interviews with individuals who declined to take part in the study took place off-site, at locations of the individual’s choosing at a time that was convenient for them.

After establishing basic rapport with potential interviewees, discussing the goal of this qualitative study, and giving them time to ask questions, we elicited oral consent to the interview and the audio recording of that interview. The interview script revolved around open-ended prompts focused on subjects’ understandings of tuberculosis as well as ‘clinical research’ in general and the DAR-901 trial specifically, including follow up questions about the feasibility of a 3-dose vaccine series. We asked participants to describe in their own words the process of being recruited to the trial, their motivations to volunteer or not, and the nature of that decision-making process. Interviews allowed for reflections on the differences in health care and medical systems between participants’ home countries and the US. In speaking with study staff, we aimed to gather their perspectives on designing and executing this study. The semi-structured interviews each took about one hour and were all conducted in English. Interviewees were given a $50 gift card as compensation for their time.^5^

As a way of gaining further contextual understanding of the trial, including processes of recruitment, we also interviewed five study investigators, including the PIs and a study coordinator. Study staff expressed interest in this qualitative study because they had been on the frontlines of subject recruitment and management of enrolled subjects, both of which were intensive. For example, for the 59 subjects enrolled in the trial, the study coordinator placed 1416 phone calls and facilitated nearly 600 patient visits over 10 months.

Upon completion of interviews, we generated transcripts and, along with additional observational field notes and debriefs between researchers, coded these for qualitative themes. We have summarized the results from each cohort based on the open and focused coding of the interview transcripts.

## Results

Table 1 summarizes basic demographics of individuals who participated in this qualitative study.

**Table 1:** Gender, Age, and Country of Origin of Interviewees

Cohort	Gender	Average Age	Country of Origin
Declined, HIV-negative	F:2 M:4	23.7	South Korea [2], Ghana [2], China, Pakistan
Enrolled, HIV-negative	F:3 M:3	32	India [3], China, Lebanon, Nigeria
Enrolled, HIV-infected	F:3 M:3	47.8	Liberia [2], Cuba, Canada, DR Congo, Australia

### To volunteer or not to volunteer?

Participants came from diverse geographic, cultural, and socioeconomic backgrounds. Education levels varied significantly, ranging from those with advanced degrees to those who had received little (less than 3 years) to no formal primary education in their home country. The annual tuberculosis incidence rates in countries of origin varied from very low (Canada 5/100,000; Australia 6/100,000) to very high (DR Congo 325/100,000; Nigeria (322/100,000) [53]. Study participants included highly educated people often employed at the parent academic institution or hospital, or students in the process of completing undergraduate or professional degrees; or people from regions of the world characterized by political-economic instability who have come to northern New England as refugees or economic migrants. The majority of individuals in the HIV-infected cohort had lived through significant experiences of uncertainty and trauma, in the context of structural inequality and/or overt political violence.

In the DAR-901 trial, nearly every encounter between participants and study staff was cross-cultural in that all study staff were US citizens who self-identified as white/Caucasian whereas most foreign-born study subjects in all cohorts identified as non-white. In the context of our qualitative study, the first and second authors were Caucasian US citizens, while the third was a foreign-born individual who recently became naturalized as a US citizen.

Our interviews revealed multiple dynamics of volunteerism and drivers of medical decision-making in relation to clinical research; and a variety of understandings of tuberculosis in general and of the DAR-901 vaccine in particular.

#### Enrolled subjects

Study subject motivations to participate in the DAR-901 Phase 1 study centred around a generalized sense of altruism, often expressed as a desire to ‘help others,’ coupled with a sense that this trial was ‘special.’ Trust in study staff, a desire to combat TB-related stigma, and financial incentives were also motivating factors. Enrolled subjects were motivated to participate in the DAR-901 trial because of the significance of DAR-901 itself, and the vaccine’s potential positive impact on global health. All enrolled participants stressed that they felt they had a specific contribution to make to *this* trial, given its location and inclusion criteria. One HIV-negative subject said there was ‘no reason *not* to participate. This [is] going to help people.’ One subject experienced an adverse skin reaction to the vaccine, but said she had no ill will towards the trial investigators because her ‘data point’ was ‘only going to help the study…I already had one scar [from BCG], and so I have another one.’

Another participant in the HIV-negative cohort said, ‘If they were recruiting just any student, I wouldn’t have been as compelled to do it just because I would have been, like, I’m sure lots of people are doing it. But there aren’t that many international students here.’ A third HIV-negative enrolled subject said her father offered to pay her *not* to do the study, presumably for fear of potential risks, but ‘I felt through doing this study, I was contributing to something significant…If you have that opportunity, you should take it, as long as it is not going to inversely impact your health and well-being…This is eventually going to help people and keep them healthy.’ Others echoed this sense that any risks of participation in the study were outweighed by the potential to contribute to ‘overcoming a huge disease burden’ as another enrolled HIV-negative participant put it. One HIV-infected individual said, ‘The danger is minimal, the benefit is tremendous. People everywhere have been kind to me, so…’.

Motivations for participation also involved conceptions of perceived direct and indirect benefit. Not all individuals we interviewed had direct experience with TB, but some did, either by having tested positive for TB exposure themselves, through relatives who had suffered from the disease, or in recognition of high mortality and morbidity from TB in their home communities. These individuals cited experience with TB as a motivation for participation in the DAR-901 trial. As one HIV-infected individual described, ‘When I was in Liberia and Ivory Coast, people died of TB. My daughter had TB…Three years ago, TB came to her.’ In some cases, enrolled subjects were motivated to participate because of perceived reduced risk of TB that exposure to the DAR-901 vaccine could offer – even though the trial was placebo-controlled, blinded and experimental. One HIV-negative subject, who had tested positive for TB exposure but decided not to take the recommended prophylactic antibiotic regimen, thought that his participation ‘may just help keep me healthy. You never know.’ Another in the HIV-infected cohort cited the growing number of new immigrants to the US (although he was also an immigrant) and the possibility for increased TB exposure. ‘I know it won’t hurt me and it might offer some protection as the world continues to change.’

Only individuals in the HIV-infected cohort cited a desire to reduce TB-related stigma as a motivation for volunteerism. While discussion of HIV serostatus was not universal – and actively avoided by some subjects – all but one HIV-infected study subject mentioned TB-related stigma as a reason to participate in the DAR-901 vaccine trial. One individual spoke about seeing how people with TB were treated in his country of origin: ‘People who were ‘shunned, ostracized…or treated like human garbage’ due to their TB status. Another individual described how her nephew died of TB and her family was affected by the stigma. She explained that despite the knowledge that TB is curable, ‘there can be whole areas of the hospital that are under quarantine but also deeply marked by stigma related to TB. People assume that if you have TB, you also have HIV.’ Even though her nephew was HIV-negative, ‘nobody would come visit him in the hospital.’ These individuals saw the possibility of an effective TB vaccine as a way to put an end to stigma as well as improve health in their countries of origin.

Enrolled interviewees described trust of study staff who also served as clinicians where they receive care as a motivation to participate. As one person put it, ‘I’m close to them. [Without a personal connection] I don’t think I would have considered it.’ Some enrolled study subjects expressed sympathy for the challenges of study recruitment as a factor motivating participation. ‘From doing clinical research myself…I know it’s sometimes really hard to recruit subjects and so that can really slow your progress.’ Those in the HIV-infected cohort were directly motivated to participate by the trust they had in their providers. As one individual put it, ‘I hope it works. I hope it makes it so I don’t get TB. I hope Dr. X makes more vaccines. He should make one for HIV.’

A stated sense of altruism and a sense that this trial was both ‘special’ and ‘important’ did not necessarily correlate with a willingness to tell others about their participation. In fact, the majority of interviewees said they discussed their enrolment in this study with either nobody or only a few people. Not all study participants told their spouse or parents that they participated. Others actively kept their enrolment from loved ones. As one HIV-negative participant said, ‘It would just be too much explain and they’d be worried or tell me not to do it because it’s not good for me.’ In another case, a HIV-infected enrolled individual said she would not share information about her participation with others, although she had enrolled in the trial**.**

Friends [possibly others who live in the region and had been approached about participating in this trial] called me to ask if I was going to participate. I said no, I am not participating….In Africa, we keep secrets. We don’t want anyone but God or the data people for this study to know our secrets.

This individual went on to describe the logics of ‘sending sickness’ [54] and how this pervaded her decision-making processes. Sharing information could render her vulnerable. Interestingly, this individual explicitly stated that her main interlocutor in the decision about participation was God. ‘God owns me, owns my life,’ she said – a comment that not only dovetailed with a stated Christian ethic of ‘helping others’ as motivation for participation but also toward much more wide-ranging aspects of her lived experience: her survival through civil war, rape, HIV infection, and immigration to the US.

Study subjects did not always receive support for participation from loved ones if they did decide to tell them. Only one interviewee mentioned his decision to enrol to everyone in his family. ‘My mom was the only one who was a bit concerned because, obviously, there is risk associated with everything. Her not being a biologist, I explained it [the idea of a booster vaccine] to her but there was still a sceptical side to her.’ One HIV-negative participant, a healthcare provider himself whose parents are both physicians in his country of birth, said that when he mentioned the possibility of his participation, they tried to dissuade him. ‘They were just fearful that there would be some side effects,’ he explained. In the end, he decided to enrol in the study but declined to tell his parents, actively denying participation when they asked. In these two cases and others, a desire to be altruistic led to deception within a social circle.

Money was a motivator for some participants, since they would earn approximately $600 for participation in the DAR-901 trial. Remuneration was especially appealing to younger subjects, such as undergraduate students, and those who were working low-paying jobs or on government assistance. Conversely, payment was a dis-incentive to others who interpreted the remuneration rate as reflective of risk. Some felt that remuneration was suspiciously high. ‘The more [the trial] pay[s], the more [the trial is] hiding something,’ said one *enrolled* individual. Others felt the trial was *not paying enough* given the amount of time that the trial demanded. One enrolled individual said, ‘I do research also… so I understand the need to recruit people and I do understand there’s not enough money to be flashing around to get people, but … if you are going to try to entice people and ask them to do a series of tests…the money incentive could have been higher.’

#### Declined subjects

In interviews with individuals who declined participation in the DAR-901 trial, they said they were simply not interested enough in the study to volunteer or that the ‘inconvenience’ and time commitment required of the trial was prohibitive. However, time was not the only factor discouraging study participation. Most individuals who declined participation said they were averse to the idea, as one person put it, of ‘adding anything unnecessary to one’s body.’ This was true even for people who had a medical and/or clinical research background. Some people who declined participation said they no longer see TB as a deadly disease or they might not have direct exposure to TB disease. Interestingly, those who voiced this reason did not give it as *their own* rationale for declining participation; rather they cited it as a rationale advanced by individuals who influenced their decision. Interviewees who declined to participate also cited scepticism about clinical research in general. Many noted concerns that the trial’s remuneration structure signalled a hidden risk in the vaccine itself.

Some speculated about how TB was transmitted, including discussing the possibility that risk of TB exposure could somehow be heightened by their participation in the trial; two individuals expressed a lack of trust in study staff. However, this was not a dominant sentiment. Indeed, one interviewee whose work schedule eventually precluded study enrolment said that trust for the clinician-scientists made him still feel ‘committed’ to the trial, even though he could not participate directly.

None of the interviewees who declined participation expressed an overt sense of fear as their reason for declining participation, but several speculated that *others* might have declined participation because of fear about the vaccine or its associated risks. Some individuals voiced general suspicion about clinical research, even if this was not directed at this trial. ‘In general, people have a lot respect for physicians, but sometimes that respect is abused…Even in the US, there have been many medical trials on minorities that will give someone pause before joining something like this.’ The subjects emphasized how important trust was in even attracting them to get more information about the trial. Without the personal connections that many subjects had to DAR-901 researchers, they would not have even considered participating in the first place.

One individual who declined to participate did so because her family forbade her out of concern for vaccine risk. ‘[I declined] because my husband won’t allow it because he thinks it was dangerous.’ When asked if she would have volunteered if her husband had not intervened, she enthusiastically said she would ‘because it helps make the vaccine better, and TB has a lot of impact on the health of developing countries like China. If this booster works, this will do good to not only the US but many countries in the world. But my husband is very stubborn.’

### Tuberculosis, vaccines, and DAR-901

Understandings of tuberculosis varied widely among those we interviewed. Highly educated individuals – both those who enrolled and those who declined – discussed disease aetiology and pathology in technical biomedical terms. Interviewees with undergraduate degree or lower education could provide a general description of the disease, with one exception. Among subjects without a medical background, a basic understanding of the disease was often tied to exposure to social media and other public health campaigns in their countries of origin. As one declined individual described, ‘We grew up learning about the Seven Killer Diseases, which are the diseases you get vaccinated for as a child. And TB is one of them.’ As another declined subject described, ‘TB is commonly referred to in movies and shows, so everyone has a general idea that the disease affected the lungs and results in coughing up blood.’ In some instances, this basic familiarity with TB and with the BCG vaccine translated into easier conversations with those in a social or medical decision-making circle about the idea of a ‘booster vaccine.’ Others found it more difficult to describe the potential benefits of this new vaccine, and knowledge of TB was more limited to a discussion of its symptoms (e.g. ‘coughing blood’) and associated social stigmas (e.g. as a sign of ‘being HIV positive’).

In response to general questions about the purpose of the DAR-901 trial recruitment criteria, all enrolled and declined individuals articulated understanding that the trial required testing this new vaccine on people who had received BCG vaccines in their countries of origin. Among the HIV-infected cohort, understanding of the trial’s goals and the science behind the administration of a vaccine booster varied widely. However, the foundational understanding that this was a vaccine which was being developed to help prevent TB was clear to all participants, as was the understanding that a new vaccine would ‘boost’ or enhance the immunity provided by the ‘old vaccine’ [BCG]. All but one subject articulated an understanding that DAR-901 was similar to BCG, but ‘hopefully better.’ The majority of interviewees were surprised to learn that BCG did not provide lifelong protection; prior to their participation in the DAR-901 trial, many had assumed that no further vaccine would be needed. ‘I feel it was very interesting… that the TB vaccine [BCG], after a certain amount of years, was no longer effective. But we never knew. Our knowledge [was] that it [BCG] is [for a] lifetime, and we never really worried.’

Individuals expressed a range of opinions about vaccines in general. Many interviewees voiced a belief in the role that vaccines play in disease prevention. Fears about vaccines did not come up as a reason to decline participation in the DAR-901 trial, but underlying medical understandings behind such decisions varied. For example, as one declined subject said, ‘Vaccines are not as dangerous as drugs because they’re helping. You prevent something. But drugs are poison to help you get rid of some condition.’ This individual indicated that they were more inclined to participate in a vaccine trial than they would have been to participate in a drug trial. Another individual in the HIV-infected cohort viewed vaccines positively: ‘I used to run to a vaccine camp when it came [to our village],’ she said, referring specifically to early childhood vaccination efforts in her country of origin. ‘I used to try to get everyone to come. People would carry babies, and I used to think that everyone should come to do the vaccines to be healthy.’

And yet, this same individual later said that her reaction was not necessarily the norm in her community, citing dynamics of fear and mistrust of biomedical authority. She used the 2014 Ebola outbreak in West Africa, and a possible Ebola vaccine, as a cautionary tale. When discussing the possibility of administering DAR-901 in her country of origin, she said, ‘Before you give, you have to explain very well. Most people will be afraid. People thought vaccines brought Ebola.’ In a similar vein, another interviewee acknowledged the association of militarized medicine with histories of colonial violence. He emphasized the ‘public relations’ work that would need to be done in ‘some countries’ to ‘combat the idea of “What are these white faces doing to us?”…You would need good education and marketing campaigns. You would have to say, “This vaccine is going to save lives.” Because anti-vaccine sentiment is something that is real on the social and political side of things.’

In contrast, most interviewees reasoned that for DAR-901 to be tested on people like them *in the US*, it must have been rigorously tested before (some specifying animal trials) and deemed safe. Many expressed a general confidence in ‘the government’ and ‘agencies that approve clinical research,’ even though most interviewees (except those involved in clinical research themselves) could not name which federal agency would need to give its approval for a new vaccine to be put into widespread clinical use.

Such comments also often led to discussions of the contrast between the US health system compared to the system they knew in their country of origin. As one participant expressed, war and political instability in her central African country has meant that ‘there is no real health care.’ This individual went on to describe how ‘you have to sometimes pawn your belongings, like a TV, to pay for the care that you need to receive in the health care system.’ She continued, ‘If you have a surgery for your appendix, you have to bring your own blood serum. Outside of the hospital setting, many people just try to treat themselves, relying on home remedies or traditional medicines.’

Many subjects mentioned schools and churches as ideal sites for administration, and felt that this should be something offered through public health care. In contrast, some noted dynamics of distrust, either saying that they would not trust the quality of the vaccine if it came from the state instead of an international nongovernmental organization. A declined HIV-negative subject noted, ‘In China…now, the hospital has become a much more economically independent unit. You have to manage and make [money]. Each hospital…has to make money and one way is through their pharmacies.’ Although most participants felt that general awareness of TB was high in their countries of origin, they also said that many probably were still under the impression that they were protected from TB if they received BCG, unless they were HIV-infected.

### Discussion: Research and lived experience, consent and culture

As our qualitatively coded data revealed, altruism, scepticism, personal commitments, self-interest and monetary incentives all contribute to the decision-making of potential research study subjects. We found this was true in the foreign-born BCG immunized patients in both the HIV-negative and HIV-infected cohorts we interviewed, and that the specifics of the clinical research question modified these dynamics. We found there was an interplay between narratives of sacrifice and those of giving back, between altruism and scepticism, as well as tensions between individual and collective sensibilities about volunteering for medical research. These dynamics were inflected by cultural sensibilities on the one hand, such as through ethno-physiological conceptions of risk [55], and experiences of structural inequality on the other, such as the lack of access to health care in a country of origin or the lived impacts war. We heed Farmer’s call ([56]: p. 47–48) not to conflate cultural difference with structural violence, including when it comes to making sense of people’s decisions about whether or not to volunteer for clinical research.

While many individuals were willing to participate in medical research, they were less willing to *talk about* their participation in clinical research with others. Altruistic expressions were coupled with a desire for privacy, concerns about what friends and family would think, or responses to others’ fears about participation. This finding reflects the ways that individual and social drivers can both compel and hinder volunteerism and complicates narratives that would equate such actions with a sense of recognized or visible social action. Altruism, while common, was also complicated by the ways some individuals tied participation in the trial to possibilities for direct personal benefit, either in the form of possible protection from TB or financial gain. Volunteerism in this context was also shaped by many enrolled participants’ perceptions of the US health care system as well as respect and trust for the physician-scientists involved in this trial, including trusted intermediaries who used social and clinical networks to make initial introductions between potential participants and study staff.

Despite a detailed informed consent form and discussion between study subjects and study staff, there was significant variation among those we interviewed about what a vaccine is and what it can and can’t do. These findings raise interesting questions about how informed consent can be influenced by cultural difference, education, socioeconomic position, and other aspects of lived experience, as others have also discussed [48, 57–62].

Several interviewees questioned whether some populations would ever be able to give fully informed consent, either because of sustained challenges of language and culture or because of broader differences in worldview and distinct social dynamics that shape the consent process. One declined individual cited the sense that ‘Asian societies have a collectivist mentality and this desire to benefit the greater good would take precedent over individual harm.’ However, this same individual (from South Korea) went on to say that ‘with modernization, Korea now has idea of western ethics so there is now a similar definition of informed consent for both countries.’ Collective decision-making practices clearly influenced many potential study subjects, either by discouraging participation or influencing disclosure of a decision to participate.

Interviewees were aware of the potential blurred boundaries between clinical research and clinical care, as they relate to the political-economy of medical research, research ethics, and the ‘outsourcing’ of clinical trials to less ‘developed’ countries. These topics came up in all but two interviews. Beyond comparisons of clinical trials which study vaccine versus drugs advanced by a corporation, interviewees spoke about how financial incentives, either to individuals or institutions in under-resourced settings, could influence research. Some interviewees described a sense of imbalance in how clinical research seemed to be conducted in their countries of origin. They expressed desires for these efforts to be more ‘ethical’ or more ‘mutually beneficial’ to the communities in which the research is conducted. One declined subject said,

In most cases, when the funding dries up or the project winds up, that [is] it. The Europeans and the Americans just walk out and are fine. Essentially, you’ve utilized [our] bodies, you’ve gained knowledge, personal, professional, and academic advancement. And, in return, [you’ve] hired a few people and built a few buildings, but [you] haven’t really given [us] anything.

Another interviewee pointed out that even if a subject population is truly informed and fully consenting, their agency could still be limited or threatened if was not protected by the judiciary in country in which research is being done. ‘If your patient population is well-educated, they know their rights. And if you have a good law enforcement system, then they can actually exercise their rights, as opposed to the patient population not knowing their rights and not necessarily understanding the risks,’ One enrolled subject described. ‘They don’t have the privilege to go sue someone. In which case, I can see [medical researchers] going in with good intentions but I can still the patient population getting exploited.’

Subjects in our study recognized that, in cases where people feel that clinical research is their only option for clinical care, true informed consent is impossible. One study staff member shared that the head of an institutional review board in a country in which he had conducted research once questioned him about ‘why Americans wasted time splitting hairs’ over the details of ethics review. As long as a trial was providing care for patients, this official argued, what did ethics review matter?

Study staff involved in this trial and in the previous SRL-172 trial in Tanzania noted that while FDA regulations are very stringent in the US, WHO regulations are less consistently enforced in Tanzania. They observed that the subject pool in Tanzania was more vulnerable than those in the US because they recognized that clinical research was connected to a source of primary care. One staff member noted, ‘While most subjects in America are looking to put gravy on their potatoes, most subjects in Tanzania are looking for the potatoes themselves.’ On this topic, one declined subject said, ‘Some people [are] waiting to die, so why not get free treatment?’ Such comments not only speak to this research versus care dynamic, but also point to differences between a vaccine trial of this type and a drug trial, even if such distinctions may at times be lost on study subjects. We summarize our coded findings in terms of the key motivations and concerns of enrolees in the tuberculosis vaccine trial in Table 2.

**Table 2:** Key motivations and concerns of enrolees in the tuberculosis vaccine trial

Coded theme	Dynamic	Response
Knowledge (direct/indirect) of tuberculosis	Many subjects were motivated to participate due to awareness of the tuberculosis epidemic and the role of tuberculosis vaccines in disease prevention	Educate potential subjects about public health problem addressed by study & how intervention aims to help people
Altruism	Subjects participated in study to aid development of tuberculosis vaccine	Mention potential for study participation to help at-risk communities
Personal relationships	Study subjects with connections to clinicians involved in study or trusted intermediaries who recommend participation were motivated to participate	Non-coercive invitation to participate in study by known clinicians where possible
Personal gain	Some subjects expect to benefit clinically from participation	Transparency about potential study benefits, if any. It is important for subjects to understand that their decision to participate in study (or not) should have no impact on quality of clinical care
Privacy	Many subjects limited release of information about study participation & its indications	Protect subject privacy in accordance with their preferences
Collective decision-making	The opinions of respected peers, including family members, influenced many approached individuals to participate or not	During consent conversation, ask potential subjects if they would like study staff to discuss study with respected peers as well (unless privacy of decision already mentioned)
Remuneration	Financial incentives to participate in the study were viewed positively by some subjects & with suspicion by others	Select a non-coercive incentive to participate in collaboration with representation from communities from which recruiting subjects
Convenience	Time required of subjects in trial can dis-incentivise participation	Be parsimonious with subject time investments; align financial incentives to them
Risk aversion	Some subjects declined out of fear the vaccine could harm them	Educate subjects about risks/benefits of study intervention & measures taken to observe & mitigate them during conduct of trial

## Conclusion

Using in-depth semi-structured interviews of declined and enrolled study subjects in the DAR-901 trial, we identified major themes influencing foreign-born individuals’ decisions about whether or not to enrol in a phase I TB vaccine clinical trial which took place in the United States. Individuals who chose to enrol in the DAR-901 trial linked a generalized sense of altruism with a very specific understanding of what this vaccine could accomplish. Study enrolees believed they were contributing meaningfully to a valid and beneficial scientific endeavour, run by people who were both qualified and cared about the participants and about improving health outcomes generally, and reducing TB mortality, morbidity, and stigma specifically. For those who declined enrolment, generalized scepticism about the motivations of physician-scientists, the ethical challenges of clinical research, and perceptions of personal risk were articulated alongside more mundane concerns about timing and effort required to participate. Financial incentives were a motivating factor for some and a locus of scepticism for others. Personal relationships between study staff and research subjects were crucial to recruitment and successful completion of the trial.

Insights from this study can help inform outreach and recruitment, informed consent, and education efforts in ongoing and future clinical trials involving foreign-born individuals. This study provides useful cross-cultural feedback at a critical moment in TB vaccine development. Our findings highlight the perceived value and need for a TB vaccine; they also identify opportunities for education about the limits of BCG. We also found that TB stigma, collective decision-making, and tailored health communications were highly influential to potential TB vaccine trial subjects. Results from these qualitative interviews reiterate the need and value of developing relationships with trusted community leaders (including clinician-scientists) as part of the research effort. These data also raise productive questions about the possibilities and limits of non-coercive incentives for participation in clinical research, and suggest the enduring importance of continued conversation about the process of conducting ethical, culturally-sensitive, and relationship-influenced science.

## Abbreviations

BCG: bacilli Calmette-Guerin (vaccine)
HIV: human immunodeficiency virus
TB: tuberculosis
US: United States
WHO: World Health Organization

## Footnotes

1 Aeras is a non-profit foundation charged with advancing the development of TB vaccines.

2 DAR-901, is an inactivated polyantigenic whole cell mycobacterial vaccine. An earlier form of DAR-901, called SRL 172, was used in a seven-year Phase III in Tanzania sponsored by the U.S. National Institutes of Health and involving adults with HIV infection who received BCG at birth. In that trial, receipt of SRL 172 was associated with significantly lower risk of microbiologically-proven tuberculosis. This trial remains the only trial in which a new tuberculosis vaccine has shown efficacy in humans. Dartmouth and the global TB vaccine research foundation Aeras collaborated to develop an improved and scalable method for manufacturing the vaccine, now designated DAR-901.

3 Dartmouth College Committee for the Protection of Human Subjects (CPHS) approved this study (CPHS #28941).

4 We had several informal phone conversations with declined HIV-infected individuals, who initially expressed interest in participating in the qualitative study. However, these individuals eventually declined, citing work schedule difficulties in one instance and some mistrust of clinical research (being ‘experimented on’) in another.

5 This level of compensation was determined in relation to what enrolled subjects were being paid for their participation in the clinical trial itself.

## References

[1] WHO End TB Strategy [Internet]. World Health Organization; 2015 [cited 2017 Jun 19]. Available from: http://www.who.int/tb/post2015_strategy/en/

[2] Dye C, Glaziou P, Floyd K, Raviglione M (2013). Prospects for tuberculosis elimination. Annu Rev Public Health.

[3] Lahey T, von Reyn CF. Mycobacterium bovis BCG and New Vaccines against Tuberculosis. In: Tuberculosis and Nontuberculous Mycobacterial Infections, Sixth Edition [Internet]. American Society of Microbiology; 2011 [cited 2017 Jun 8]. p. 162–181. Available from: http://www.asmscience.org/content/book/10.1128/9781555817138.ch10

[4] Knight GM, Griffiths UK, Sumner T, Laurence YV, Gheorghe A, Vassall A (2014). Impact and cost-effectiveness of new tuberculosis vaccines in low-and middle-income countries. Proc Natl Acad Sci.

[5] The Global Pipeline of TB Vaccine Candidates [Internet]. Aeras. [cited 2017 Jun 8]. Available from: http://www.aeras.org/pages/portfolio-approach

[6] Kaufmann SH, Weiner 3rd J, Maertzdorf J. Accelerating tuberculosis vaccine trials with diagnostic and prognostic biomarkers. Expert Rev Vaccines [Internet]. 2017 [cited 2017 Jul 19];(just-accepted). Available from: http://www.tandfonline.com/doi/abs/10.1080/14760584.2017.1341316 10.1080/14760584.2017.1341316 28608729

[7] von Reyn CF, Mtei L, Arbeit RD, Waddell R, Cole B, Mackenzie T (2010). Prevention of tuberculosis in Bacille Calmette–Guérin-primed, HIV-infected adults boosted with an inactivated whole-cell mycobacterial vaccine. AIDS.

[8] von Reyn CF, Lahey T, Arbeit RD, Landry B, Kailani L, Adams LV (2017). Safety and immunogenicity of an inactivated whole cell tuberculosis vaccine booster in adults primed with BCG: a randomized, controlled trial of DAR-901. PLoS One.

[9] Verheggen FWSM, Nieman F, Jonkers R (1998). Determinants of patient participation in clinical studies requiring informed consent: why patients enter a clinical trial. Patient Educ Couns.

[10] Locock LSL (2011). Personal benefit, or benefiting others? Deciding whether to take part in clinical trials. Clinical Trials London, England.

[11] Wright JRWT, Schiff S, Dubois S, Crooks D, Haines PT (2004). Why cancer patients enter randomized clinical trials: exploring the factors that influence their decision. J Clin Oncol.

[12] Dainesi SM, Goldbaum M (2014). Reasons behind the participation in biomedical research: a brief review. Rev Bras Epidemiol.

[13] Nappo SIG, Sanchez ZM. Motives for participating in a clinical research trial: a pilot study in Brazil. BMC Public Health. 2013;13(19) 10.1186/1471-2458-13-19 PMC3554489 23302375

[14] Mtunthama NMR, French N, Molyneux ME, Zijlstra EE, Gordon SB (2008). Malawians permit research bronchoscopy due to perceived need for healthcare. J Med Ethics.

[15] Zammar GMH, Shah J, Phadtare A, Cofiel L, Pietrobon R. So different, yet so similar: meta-analysis and policy modeling of willingness to participate in clinical trials among Brazilians and Indians. PLoS One. 2010;5(12):e14368. https://doi.org/10.1371/journal.pone.0014368. 10.1371/journal.pone.0014368 PMC3002940 21179556

[16] Tarimo EAMTA, Kohi TW, Bakari M, Mhalu F, Kulane A. Reasons for declining to enroll in a phase O and II HIV vaccine trails after randomization among eligible volunteers in Dar es Slaam, Tanzania. PLoS One. 2011;6(2):e14619. https://doi.org/10.1371/journal.pone.0014619. 10.1371/journal.pone.0014619 PMC3040178 21358826

[17] Marshall PA, Koenig BA, Grifhorst P, Van Ewijk M. Ethical issues in immigrant health care and clinical research. In: Handbook of immigrant health [Internet]. Springer; 1998 [cited 2017 Jun 8]. p. 203–226. Available from: http://link.springer.com/10.1007/978-1-4899-1936-6_11

[18] Khan K, Muennig P, Behta M, Zivin JG (2002). Global drug-resistance patterns and the management of latent tuberculosis infection in immigrants to the United States. N Engl J Med.

[19] Cain KP, Benoit SR, Winston CA, Mac Kenzie WR (2008). Tuberculosis among foreign-born persons in the United States. JAMA.

[20] Petryna A. When experiments travel: clinical trials and the global search for human subjects: Princeton University Press; 2009.

[21] Thiers FA, Sinskey AJ, Berndt ER (2008). Trends in the globalization of clinical trials. Nat Rev Drug Discov.

[22] Truong THWJ, Cook EF, Joffe S (2011). Altruism among participants in cancer clinical trials. Clin Trials.

[23] McCann SKCMK, Entwistle VA. Reasons for participating in randomized controlled trials: conditional altruism and considerations for self. Trials. 2010;11 10.1186/1745-6215-11-31 PMC2848220 20307273

[24] Llewellyn-Thomas HA, McGreal MJ, Thiel EC, Fine S, Erlichman C (1991). Patients' willingness to enter clinical trials: measuring the association with perceived benefit and preference for decision participation. Soc Sci Med.

[25] Schutta KM, Burnett CB. Factors that influence a patient’s decision to participate in a phase I cancer clinical trial. In: Oncology Nursing Forum [Internet]. 2000 [cited 2017 Jun 8]. p. 1435–1438. Available from: http://europepmc.org/abstract/med/11058975 11058975

[26] Jain SL. Malignant: how cancer becomes us: Univ of California Press; 2013.

[27] Lowther K, Harding R, Ahmed A, Gikaara N, Ali Z, Kariuki H (2016). Conducting experimental research in marginalised populations: clinical and methodological implications from a mixed-methods randomised controlled trial in Kenya. AIDS Care.

[28] Hall S, Goddard C, Speck PW, Martin P, Higginson IJ (2013). “It makes you feel that somebody is out there caring”: a qualitative study of intervention and control participants’ perceptions of the benefits of taking part in an evaluation of dignity therapy for people with advanced Cancer. J Pain Symptom Manag.

[29] Sachs B (2010). The exceptional ethics of the investigator-subject relationship. J Medicine and Philosophy.

[30] Sahay S, Kumar M, Srikrishnan AK, Ramanathan V, Mehendale S (2014). Experiences in recruiting volunteers through community-based initiatives in Phase-1 vaccine trials in India. Hum Vaccines Immunother.

[31] Bigorra J, Banos JE (1990). Weight of financial reward in the decision by medical students and experienced healthy volunteers to participate in clinical trials. Eur J Clin Pharmacol.

[32] Stunkel LGC (2011). More than money: a review of the literature examining healthy volunteer motivations. Contemporary Clinical Trials.

[33] Russell ML, Moralejo DG, Burgess ED (2000). Paying research subjects: participants perspectives. J Med Ethics.

[34] Reed E, Fisher CB, Blakenship KM, West BS, Koshnood K (2017). Why female sex workers participate in HIV research: the illusion of voluntariness. AIDS Care.

[35] Tishler CL, Bartholomae S (2002). The recruitment of normal healthy volunteers: a review of the literature on the use of financial incentives. J Clin Pharmacol.

[36] Vere DW (1991). Payments to healthy volunteers-ethical problems. Br J Clin Pharmacol.

[37] Donald R, Hoover BCEAM (2000). Attitudes of adolescents/young adult women toward human papilloma virus vaccination and clinical trials. Health Care for Women International.

[38] Erves JC, Mayo-Gamble TL, Hull PC, Duke L, Miller ST (2017). Adolescent participation in HPV vaccine clinical trials: are parents willing?. J Community Health.

[39] Brintnall-Karabelas JSS, Cadman ME, Squires C, Whorton K, Pao M (2011). Improving recruitment in clinical trials: why eligible participants decline. J Empir Res Hum Res Ethics.

[40] Corbie-Smith G, Thomas SB, George DMMS (2002). Distrust, race, and research. Arch Intern Med.

[41] Igwe E, Woodburn J, Davolos J (2016). Patient perceptions and willingness to participate in clinical trials. Gynecol Oncol.

[42] Lock M (2010). Nguyen V-K.

[43] Wong YN (2013). Shared benefits of clinical research should come with shared responsibility.

[44] Masiye FKN, Hyder A, Ndebele P, Mfutso-Bengo J (2008). Why mothers choose to enroll their children in malaria clinical studies and the involvement of relatives in decision making: evidence from Malawi. Malawi Med J.

[45] Annas GJ (2009). Globalized clinical trials and informed consent. N Engl J Med.

[46] Glickman SW, McHutchison JG, Peterson ED, Cairns CB, Harrington RA, Califf RM, et al. Ethical and scientific implications of the globalization of clinical research [Internet]. Mass Medical Soc; 2009 [cited 2017 Jun 8]. Available from: http://www.nejm.org/doi/full/10.1056/NEJMsb0803929 10.1056/NEJMsb0803929 19228627

[47] Shapiro HT, Meslin EM. Ethical issues in the design and conduct of clinical trials in developing countries [Internet]. Mass Medical Soc; 2001 [cited 2017 Jul 19]. Available from: http://www.nejm.org/doi/pdf/10.1056/NEJM200107123450212. 10.1056/NEJM200107123450212 11450665

[48] Adams V, Miller S, Craig S, Le PV, Varner M (2007). Others. Informed consent in cross-cultural perspective: clinical research in the Tibetan autonomous region, PRC. Cult Med Psychiatry.

[49] Regmi PR, Aryal N, Kurmi O, Pant PR, Teijlingen E, Wasti SP. Informed Consent in Health Research: Challenges and Barriers in Low-and Middle-Income Countries with Specific Reference to Nepal. Dev World Bioeth [Internet]. 2016 [cited 2017 Jul 19]; Available from: http://onlinelibrary.wiley.com/doi/10.1111/dewb.12123/full. 10.1111/dewb.12123 27518590

[50] Dawson L, Kass NE (2005). Views of US researchers about informed consent in international collaborative research. Soc Sci Med.

[51] Sankar P (2004). Communication and miscommunication in informed consent to research. Med Anthropol Q.

[52] Steinke EE (2004). Research ethics, informed consent, and participant recruitment. Clin Nurse Spec.

[53] WHO Global Tuberculosis Report [Internet]. World Health Organization; 2016 [cited 2017 Jun 5]. Available from: http://www.who.int/tb/publications/global_report/en/

[54] Farmer P (1990). Sending sickness: sorcery, politics, and changing concepts of AIDS in rural Haiti. Med Anthropol Q.

[55] Nichter M. Global health: why cultural perceptions, social representations, and biopolitics matter: University of Arizona Press; 2008.

[56] Farmer P (2004). Pathologies of power: health, human rights, and the new war on the poor. Vol. 4. Univ of California press.

[57] Barata PC, Gucciardi E, Ahmad F, Stewart DE (2006). Cross-cultural perspectives on research participation and informed consent. Soc Sci Med.

[58] Shafiq N, Malhotra S (2011). Ethics in clinical research: need for assessing comprehension of informed consent form?. Contemp Clin Trials.

[59] Vallely A, Lees S, Shagi C, Kasindi S, Soteli S, Kavit N (2010). How informed is consent in vulnerable populations? Experience using a continuous consent process during the MDP301 vaginal microbicide trial in Mwanza, Tanzania. BMC Med Ethics.

[60] Molyneux CS, Peshu N, Marsh K (2005). Trust and informed consent: insights from community members on the Kenyan coast. Soc Sci Med.

[61] Fitzgerald DW, Marotte C, Verdier RI, Johnson WD, Pape JW (2002). Comprehension during informed consent in a less-developed country. Lancet.

[62] Kripalani S, Bengtzen R, Henderson LE, Jacobson TA (2008). Clinical research in low-literacy populations: using teach-back to assess comprehension of informed consent and privacy information. IRB Ethics Hum Res.

